# Exploring Technology Perceptions and Intentions to Use in Older Adults

**DOI:** 10.1093/geroni/igaa057.627

**Published:** 2020-12-16

**Authors:** Stephanie Williams, Elizabeth Orsega-Smith, Laurie Ruggiero

**Affiliations:** 1 Discover, New Castle, Delaware, United States; 2 University of Delaware, Newark, Delaware, United States

## Abstract

By the year 2035, the older adult population is expected to expand to 78 million in the United States. Advancing technology has made aging in place a more accessible possibility; however, understanding what is preventing this population from adopting the advancing devices remains to be a challenge as the presence of a digital divide continues to exist. A 34-question survey adapted from the Technology Proficiency Self-Assessment Questionnaire, and the National Technology Readiness survey was administered to 101 participants over the age of 50 across five local senior centers. The average age range was 70-79 and most were female (79.2%), white (69%), and owned or had access to technology such as a computer or cell phone (93%). Examples of findings include 86% felt technology limited human interaction and 69% felt the use of technology could lead to security risk and a breach of privacy, while 79% felt technology could improve their quality of life. Results found 60-69-year-olds were significantly more likely (p<.05) to have or use technology versus 80-89-year-olds. Correlation between perception and intent to use technology among older adults was positive with a coefficient value of .59(p<.01). Showing a relationship between perceptions and behavioral intentions to use technology, specifically in 60-69-year-olds. This study found access to technology (i.e. computers, cell phones, internet) was not a driving factor of usage among the older population attending a senior center. To increase understanding further exploration of perceptions and intentions to use technology is warranted in the older adult population.

